# Medical College of Georgia, at Augusta

**Published:** 1857-10

**Authors:** 


### Medical College of Georgia at Augusta.

Circumstances (unnecessary to detail) make it proper for us to say, that our relations with the Medical College of Georgia, are and have been, of the most friendly character, and in addition to this, it is with pleasure, that we would say to our friends, who design attending Medical Lectures, that they would scarcely do better than to spend the winter in Augusta, where they will find an institution of high, deservedly high, character, and with a Faculty, perhaps, inferior to none in the Union.

It is very true that the Institution is so well known that it needs no compliment from us, but to correct a misapprehension, we feel it to be our duty, in *simple justice* to the Institution, to say this much.

